# Radiological and Biochemical Diagnosis of a Primary Mediastinal Germ Cell Tumour: A Case Report

**DOI:** 10.7759/cureus.112137

**Published:** 2026-07-06

**Authors:** Hozaifa Sahi, Divneet Kaur Chadha, Ivo Hennig

**Affiliations:** 1 Medicine, Glan Clwyd Hospital, Rhyl, GBR; 2 Medical Oncology, Clatterbridge Cancer Centre NHS Foundation Trust, Liverpool, GBR

**Keywords:** anterior mediastinal mass, cisplatin etoposide chemotherapy, ct thorax, mediastinal germ cell tumor, ultrasound guided transthoracic mediastinal biopsy

## Abstract

Anterior mediastinal masses pose a diagnostic challenge due to the non-specific presentation and overlapping clinical features, which may prolong investigations and delay diagnosis. This case describes a previously fit and well young man who presented with symptoms initially suspected to be caused by a respiratory infection.

Following referral from primary care to the emergency department for non-improving respiratory symptoms, a chest X-ray demonstrated a large abnormality in the left hemithorax. A subsequent contrast-enhanced CT thorax revealed a large anterior mediastinal mass causing significant compression of the left lung, with associated pulmonary nodules suspicious for metastatic disease. Serum tumour markers demonstrated a markedly elevated alpha-fetoprotein (AFP), with moderate elevation of lactate dehydrogenase (LDH) and beta-human chorionic gonadotrophin (β-hCG). A CT abdomen and pelvis showed no further disease, and a testicular ultrasound demonstrated no focal testicular abnormality, supporting a primary mediastinal origin of the mass.

The combined radiological and biochemical findings were diagnostic of a primary mediastinal non-seminomatous germ cell tumour, allowing urgent oncological treatment with cisplatin and etoposide chemotherapy to be initiated prior to final histopathological confirmation. A tissue sample obtained via ultrasound-guided biopsy confirmed the primary lesion to be a pleomorphic, yolk-sac tumour. The patient was then subsequently transferred to a tertiary specialist centre where he was treated with curative-intent carboplatin, bleomycin, vincristine, cisplatin and etoposide (CBOP-BEP) chemotherapy. A promising biochemical response to initial management was demonstrated with a decline in tumour markers, namely, AFP.

This case highlights the increasingly central role of integrating multimodal imaging with serum tumour markers in the assessment of complex anterior mediastinal masses, enabling prompt oncological decision-making in aggressive disease.

## Introduction

Anterior mediastinal masses present a diagnostic challenge due to the variety in aetiology and severity. Differentials include a broad range of both benign and malignant conditions. Differential diagnoses are traditionally approached using the ‘4 Ts’ framework: thymic lesions, teratomas, thyroid lesions, and ‘terrible’ lymphomas [1]. The proximity of these lesions to critical thoracic structures, coupled with the underlying pathological processes, often determines both clinical presentation and disease severity.

Germ cell tumours (GCTs) are the most frequently occurring neoplasms in adolescents and most commonly arise from the gonads [2]. Extragonadal germ cell tumours are rare and are thought to arise from the improper migration and retention of germ cell precursors during embryogenesis [2]. The mediastinum is the most common extragonadal location and predominantly affects young male patients. Primary mediastinal germ cell tumours (PMGCTs) account for approximately 1-5% of all germ cell tumours and can be broadly classified into seminomatous and non-seminomatous tumours [3]. Non-seminomatous subtypes include yolk sac tumours, embryonal carcinomas, choriocarcinomas, post-pubertal teratomas and mixed germ cell tumours containing both seminomatous and non-seminomatous elements [4]. Non-seminomatous PMGCTs are associated with more aggressive biological behaviour and poorer prognosis than seminomatous tumours.

This case report discusses a young man diagnosed with a primary anterior mediastinal pleomorphic yolk sac tumour following chest X-ray, CT imaging, tumour marker assessment, testicular ultrasound, and ultrasound-guided biopsy.

## Case presentation

A previously fit and well man in his mid-twenties presented to his general practitioner (GP) with a persistent cough on a background of progressive shortness of breath, fatigue, and declining exercise tolerance. His symptoms had become increasingly noticeable during his training to become a Marine. He was initially treated for a presumed respiratory infection with a course of antibiotics. However, his symptoms did not improve, causing him to re-present to his GP.

On reassessment, he was noted to be tachycardic and tachypnoeic, with reduced air entry bilaterally on chest auscultation. He was therefore referred urgently to secondary care for a chest X-ray. In the emergency department, blood tests showed mild leukocytosis of 11.5 × 10⁹/L (4-11 × 10⁹/L), with normal neutrophils. His C-reactive protein (CRP) was elevated at 93 mg/L (<5 mg/L).

**Figure 1:**
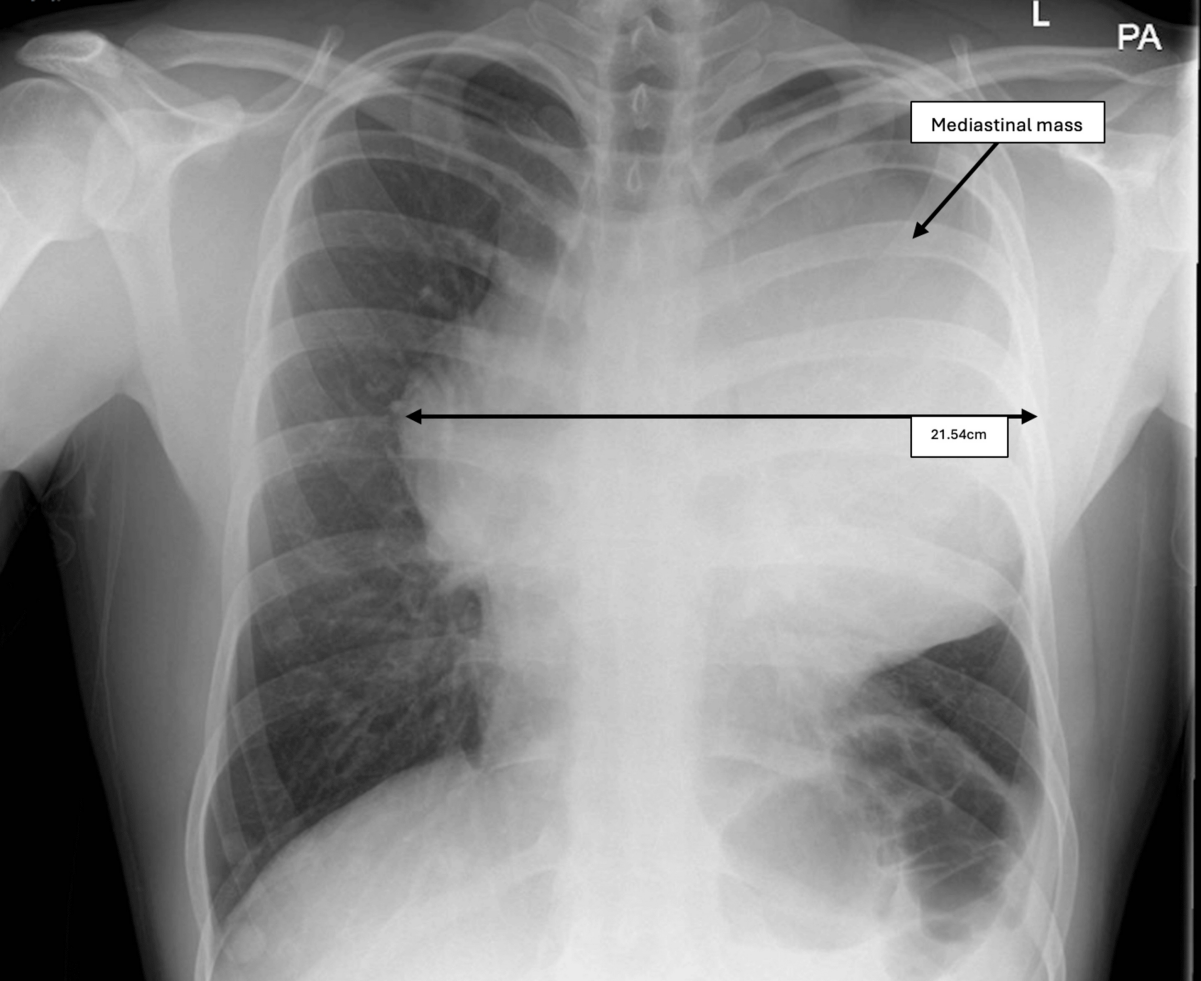
Posteroanterior chest radiograph demonstrating marked widening of the upper mediastinal contours with a large homogeneous opacity projected over the left upper and mid zones The black arrow indicates the superior aspect of the mediastinal mass. The double-headed arrow denotes the transverse diameter of the mediastinal mass. The appearances prompted urgent cross-sectional imaging for further characterisation.

The chest X-ray demonstrated marked widening of the upper mediastinal contours, with a large homogeneous opacity overlying the left upper and mid lung zones (Figure 1). It was also noted that there was a small round focal density in the right lung base overlying the eighth rib and another small round density just beneath the ninth rib, raising suspicion of metastatic disease.

**Figure 2:**
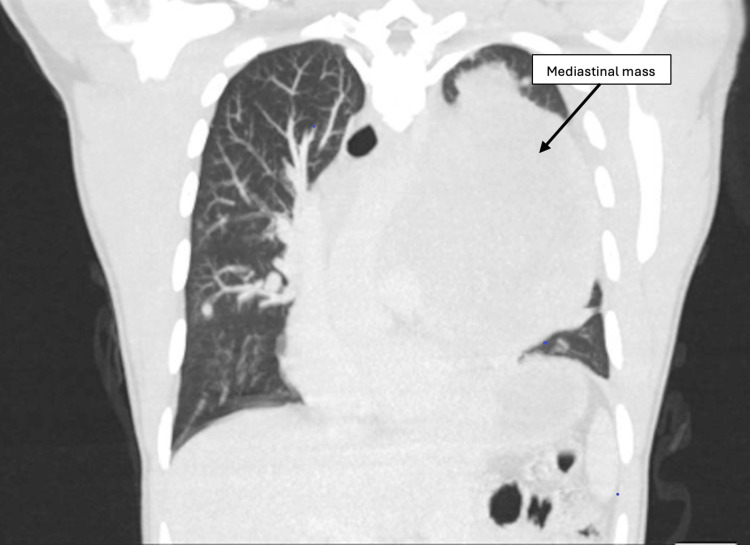
Coronal contrast-enhanced CT thorax demonstrating a large heterogeneous anterior mediastinal mass occupying much of the left hemithorax, with marked compression of the left lung and rightward mediastinal shift The mass shows contact with the pleura and chest wall. The black arrow indicates the anterior mediastinal mass.

Given the concerning findings on the chest X-ray, he subsequently had a CT scan of the thorax on the same day, which showed a large heterogeneously enhancing mass involving the anterior mediastinum extending superiorly into the prevascular compartment and right-sided tracheal and mediastinal shift (Figure 2).

**Figure 3:**
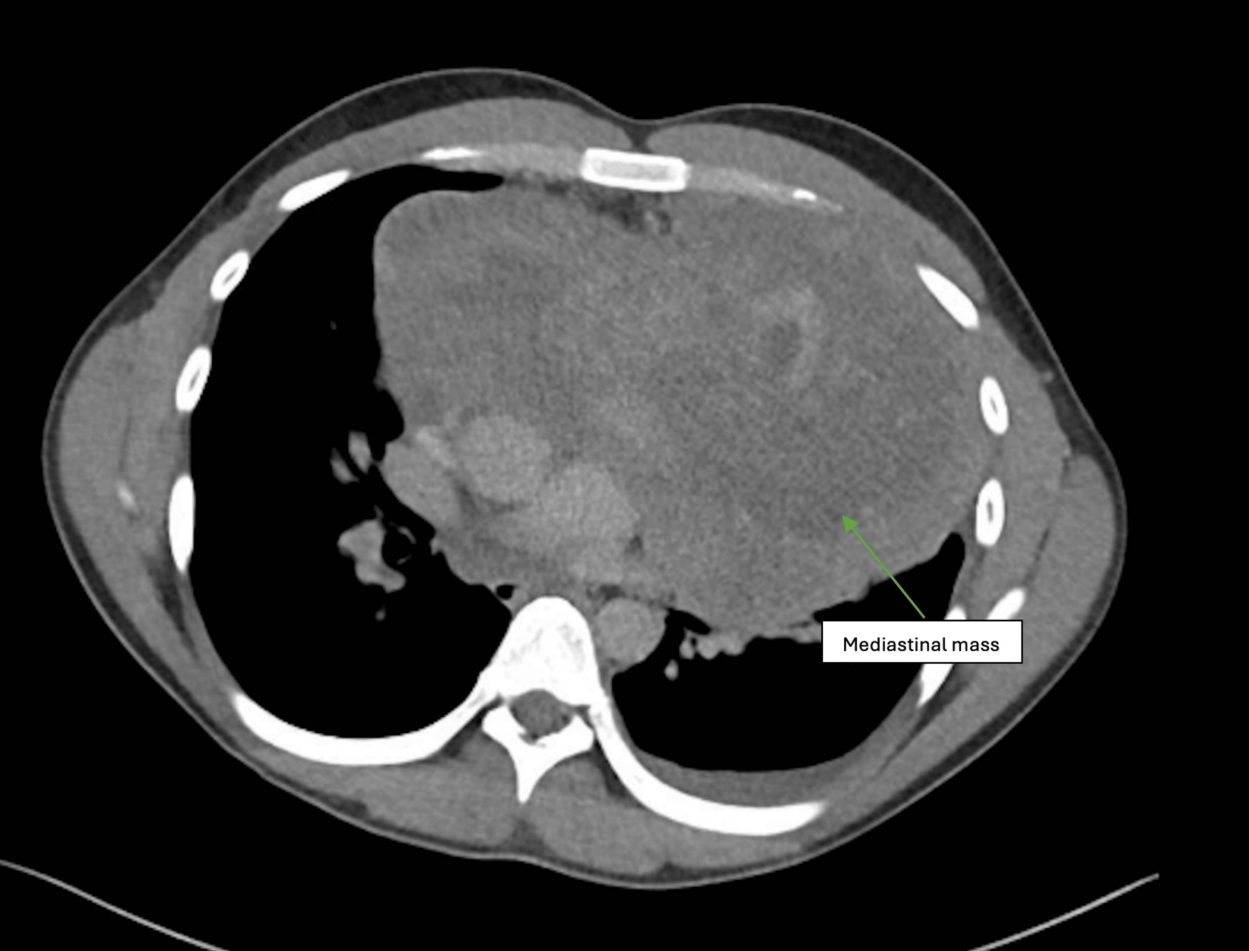
Axial contrast-enhanced CT thorax showing the anterior mediastinal mass bordering the pericardium, with associated left pleural effusion The appearances raised concern for local involvement and contributed to staging and treatment planning. The arrow highlights the anterior mediastinal mass, adjacent to the pericardium.

There was radiological evidence of involvement of the lateral chest wall, the left pleura, and the pericardium associated with mild pericardial effusion (Figure 3). The superior vena cava was compressed and displaced, although it was still patent. Additionally, bilateral metastatic lung deposits were seen. The CT was suggestive of a metastatic malignant anterior mediastinal lesion, likely a malignant GCT, given the patient's age and presentation.

Tumour markers were requested following the CT findings. AFP was markedly elevated at >50,000 kU/L (1-6 kU/L), with a mildly raised β-hCG at 17 IU/L (<2 IU/L) and LDH at 487 U/L (<250 U/L). A staging CT scan of the abdomen and pelvis did not demonstrate abdominal or pelvic metastatic disease, and there were no signs of retroperitoneal lymphadenopathy. An ultrasound of the testes showed no focal intratesticular lesions, supporting a primary mediastinal origin rather than a gonadal primary of the mass. As the anterior mediastinal mass was in direct contact with the chest wall, an ultrasound-guided biopsy was performed.

**Figure 4:**
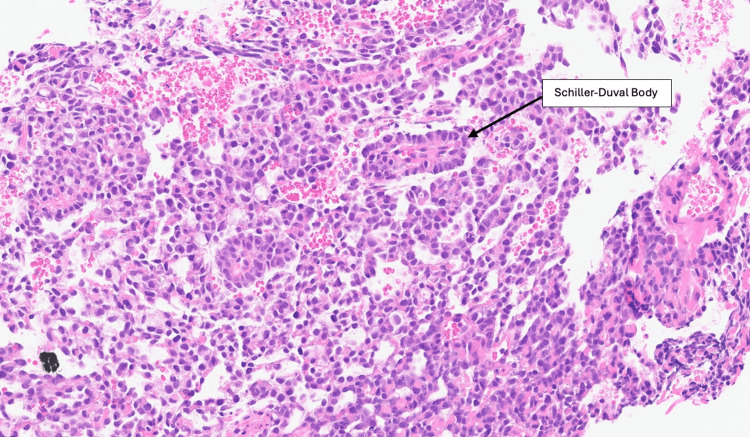
Hematoxylin and eosin (H&E) stain showing plasmacytoid appearance of cells with Schiller-Duval bodies that are pathognomonic for yolk sac tumours

**Figure 5:**
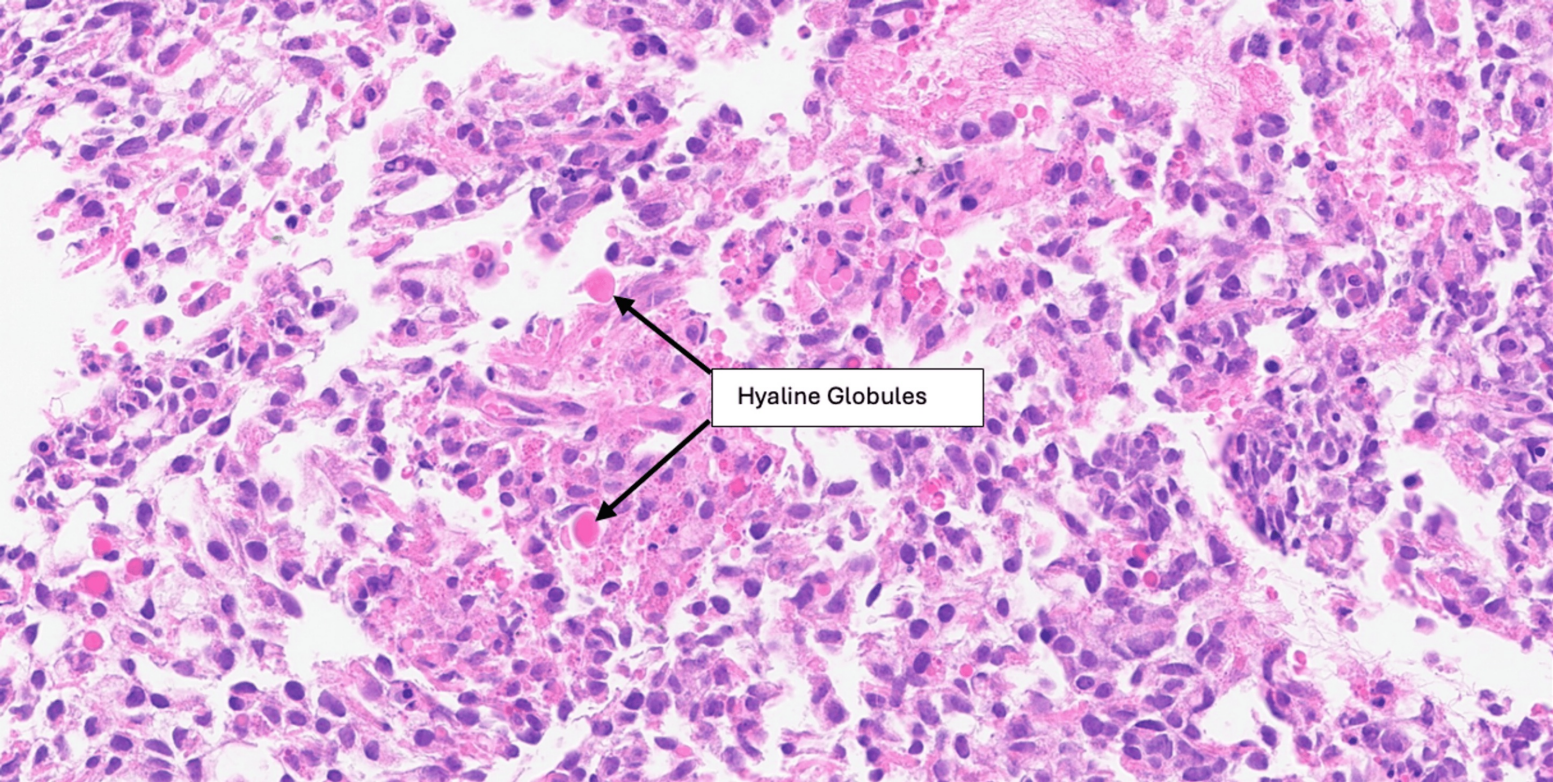
H&E stain showing hyaline globules supporting the diagnosis of a yolk sac tumour

**Figure 6:**
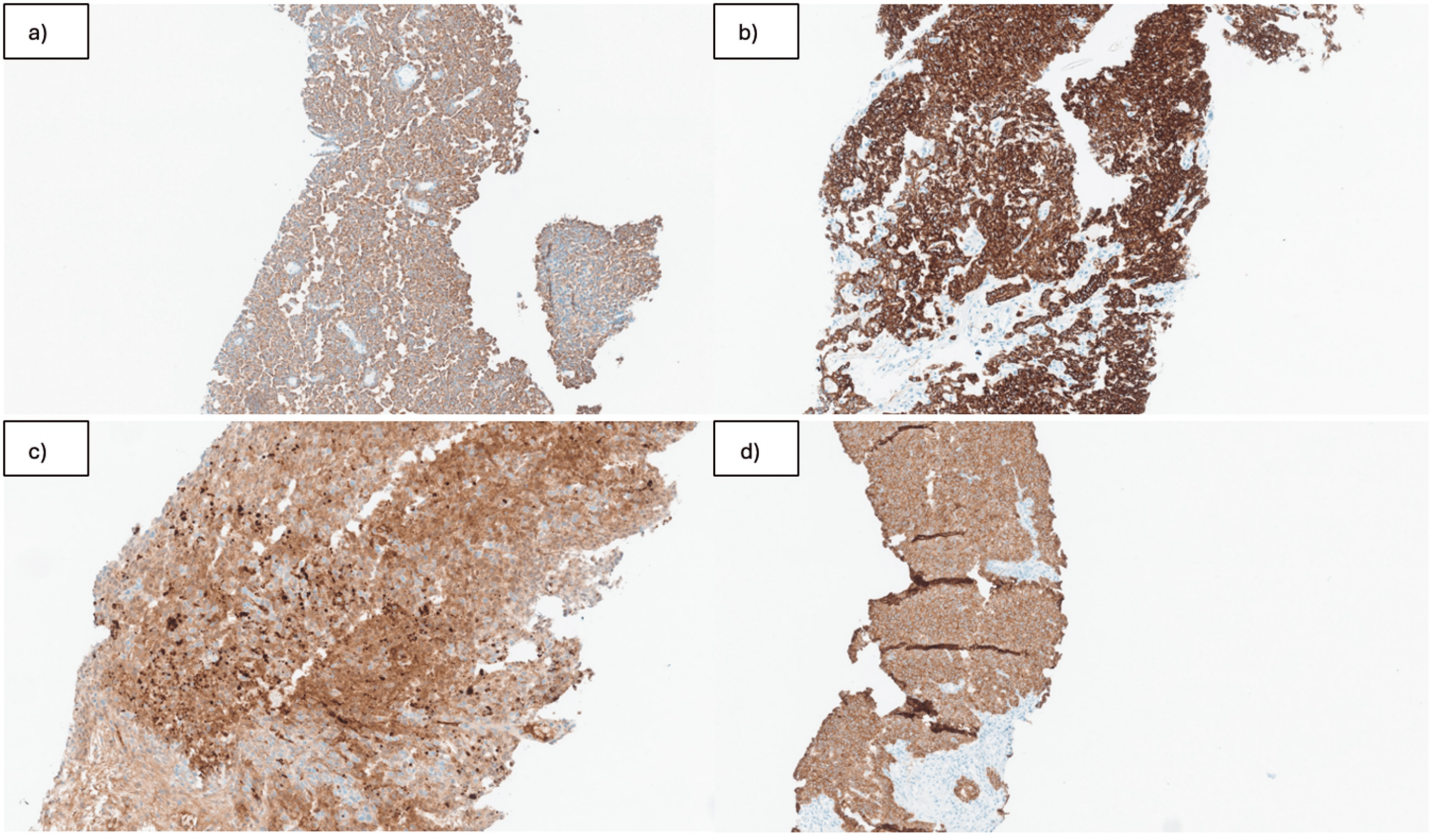
Immunohistochemistry stains showing positive uptake for a) MNF116, b) CAM5.2, c) AFP (specific for yolk sac tumours) and d) CD117

The combination of these imaging findings and the markedly elevated AFP was diagnostic for a primary mediastinal non-seminomatous germ cell tumour, although the subtype was unknown at the time. In view of the aggressive disease burden and clinical presentation, the patient was commenced on etoposide and cisplatin chemotherapy locally prior to final histopathological and immunohistochemical confirmation becoming available. Later, the biopsy results from the mass showed a plasmacytoid appearance of the cells and Schiller-Duval bodies (Figure 4). Hyaline globules were also noted on biopsy (Figure 5). Immunohistochemistry of the cells showed diffuse positivity of CAM5.2, MNF116, AFP and CD117 (Figure 6), which confirmed a diagnosis of a primary mediastinal pleomorphic yolk sac tumour.

Due to the high-risk nature of the PMGCT, he was subsequently transferred to a tertiary specialist cancer centre for curative intent carboplatin, bleomycin, vincristine, cisplatin and etoposide (CBOP-BEP) chemotherapy. Fertility preservation was undertaken with sperm banking following the first cycle of treatment. The patient remains under specialist oncological follow-up, with serial tumour marker monitoring and planned post-treatment CT imaging to assess treatment response.

## Discussion

In a majority of cases, patients with anterior mediastinal masses present symptomatically [5]. However, incidental findings in those who are asymptomatic do occur. Clinical presentation is often non-specific, with symptoms such as cough, dyspnoea, chest discomfort and fatigue. Hoarseness, haemoptysis and dysphagia may suggest more advanced disease, particularly where there is compression or invasion of adjacent mediastinal structures.

The initial investigation of anterior mediastinal masses requires adequate radiological assessment to ensure clear anatomical localisation and differentiation based on their predominant density pattern [6]. Chest X-rays reveal the first indication of a mediastinal abnormality. However, they are limited in further characterisation of the mass. Recent multidisciplinary guidance from the British Thoracic Oncology Group (BTOG) Thymic Malignancies Special Interest Group identifies contrast-enhanced CT as the primary imaging modality in the assessment of anterior mediastinal lesions [7]. MRI scans tend to be reserved for selected cases where additional tissue characterisation may aid diagnostic clarification, specifically, their internal composition, mass effect, local invasion and metastatic disease.

Masses in the anterior mediastinum are traditionally characterised using the ‘4Ts’ framework; thymic lesions, teratomas, thyroid lesions, and 'terrible' lymphomas [1]. However, this must be clinically correlated with the patient’s demographics, presentation, radiological findings and biochemical markers to come to a unifying diagnosis.

In the present case, a diagnosis of lymphoma was a pertinent differential to consider, given the patient’s age and respiratory symptoms. Importantly, lymphomas represent the most common mediastinal mass in the paediatric population, accounting for approximately 50% of cases [8]. Mediastinal lymphomas are broadly divided into Hodgkin and non-Hodgkin subtypes, with the former being more common [9]. On imaging, characteristic features of a Hodgkin lymphoma in the anterior mediastinum include irregular contours, surface lobulation, an absence of vascular involvement and a high prevalence of associated mediastinal lymphadenopathy [10]. However, the markedly elevated AFP would rule out a lymphoma being the primary lesion.

Thymic malignancy was also considered because of the location of the mass in the anterior mediastinum. On CT scans, thymomas typically present as an encapsulated soft tissue lesion and demonstrate homogeneous attenuation with contrast enhancement [11]. In advanced disease, dissemination to the pleural or pericardial space may also occur, known as drop metastases. In this case, while the CT thorax seemed to indicate pleuropericardial involvement, the patient’s young age and tumour marker profile effectively excluded thymoma as the primary diagnosis.

A malignant mediastinal GCT became the leading differential diagnosis after correlation of imaging and biochemical findings. GCTs account for 10-15% of anterior mediastinal masses and are broadly classified into seminomatous and non-seminomatous subtypes [6]. Of these, non-seminomas account for 60% of all PMGCTs, with mature teratomas being the most frequently encountered histology, which tend to have better prognostic outcomes [12]. On CT imaging, teratomas most commonly appear as a well-defined unilocular or multilocular cystic lesion containing fluid, soft tissue and fat attenuation [13]. Comparatively, radiological findings for other non-seminomatous germ cell tumours (NSGCTs) include large, bulky, ill-circumscribed masses with lobulated contours [14]. Due to their rapid growth, invasion of adjacent structures and distant metastasis may occur by the time of presentation. Pleural and pericardial effusions are also common. NSGCTs include yolk sac tumours, embryonal carcinomas, choriocarcinomas, and mixed germ cell tumours, which contain both seminomatous and non-seminomatous histological components. In all cases, a testicular ultrasound is required to exclude a gonadal primary, while a CT abdomen and pelvis assists with staging and assessment for visceral metastatic disease [15].

In this case, the combination of imaging findings and the tumour marker profile confirmed the diagnosis of a malignant mediastinal non-seminomatous germ cell tumour. Although histopathological confirmation remains important, treatment may be initiated, where safely feasible, prior to final tissue diagnosis in clinically aggressive presentations with characteristic radiological findings and markedly elevated serum tumour markers. Imaging-guided percutaneous needle biopsy remains a safe and effective method of obtaining tissue diagnosis from mediastinal masses [16]. In this case, ultrasound-guided biopsy was appropriate as the anterior mediastinal mass directly abutted the chest wall, providing an accessible acoustic window for sampling.

Ultrasound-guided biopsy offers several practical advantages, namely the avoidance of exposure to ionising radiation and real-time needle visualisation, allowing for dynamic needle adjustment during procedures [17]. Importantly, the use of Doppler can help avoid adjacent vascular structures. CT-guided biopsy is better suited for deeper mediastinal lesions or when there is no safe sonographic window, as it allows for superior anatomical localisation and needle-path planning [18]. During the biopsy, the most important complications to consider are pneumothorax and haemorrhage.

Yolk sac tumours demonstrate histological variability, with the reticular or microcystic pattern being the most commonly described appearance. Other recognised growth patterns include vitelline, pseudopapillary, solid, spindle cell, myxomatous, macrocystic, intestinal-like and hepatoid patterns [19]. Immunohistochemistry plays a central role in the diagnosis of yolk sac tumours and should be interpreted alongside tumour morphology and the wider immunophenotypic profile. AFP remains one of the most widely used markers in routine practice [20]. CD117 expression and intracytoplasmic hyaline globules are recognised supportive features of yolk sac tumour histology. Schiller-Duval bodies remain a pathognomonic feature of yolk sac tumours when identified [19].

In the management of PMGCTs, cisplatin-based chemotherapy remains the cornerstone of treatment, with common first-line regimens including BEP (bleomycin, etoposide and cisplatin) and VIP (etoposide, ifosfamide and cisplatin) [12]. Intensified induction regimens such as CBOP-BEP have also been used in patients with poor-prognosis metastatic non-seminomatous germ cell tumours, including PMGCTs, as per the International Germ Cell Cancer Collaborative Group’s (IGCCCG) risk group classification. Favourable response rates have been demonstrated, with 74% of patients treated with CBOP-BEP showing complete or partial response with normal tumour markers, compared with 61% for standard BEP therapy [21].

In the current case, the patient was initially commenced on etoposide and cisplatin prior to escalation to curative-intent CBOP-BEP chemotherapy at a tertiary specialist centre. This initial approach reflected the need for urgent cytoreduction in the context of extensive thoracic disease burden and respiratory compromise, whilst avoiding early bleomycin exposure before specialist assessment. Although bleomycin-containing regimens, such as BEP, remain standard first-line therapy for poor-prognosis metastatic NSGCTs, bleomycin can cause clinically significant pulmonary toxicity and therefore requires careful consideration in patients with compromised respiratory reserve [22].

Primary surgical resection is generally not favoured in mediastinal germ cell tumours, given their tendency to present with locally invasive or metastatic disease, except in cases of mature teratomas, as they do not respond to chemotherapy [12,23]. Nevertheless, following chemotherapy, surgical resection has an important role in selected patients with residual or recurrent disease.

## Conclusions

Primary mediastinal yolk sac tumours are rare and aggressive malignancies that may present with non-specific respiratory symptoms. This case highlights the importance of synthesising multi-modality imaging with serum tumour markers and immunochemistry in the assessment of anterior mediastinal masses. The approach demonstrated in this case ensured prompt detection of a rare neoplasm and expedited specialist oncological management.

## References

[1] Almeida PT, Heller D (2024). Anterior mediastinal mass. StatPearls [Internet].

[2] El-Zaatari ZM, Ro JY (2021). Mediastinal germ cell tumors: a review and update on pathologic, clinical, and molecular features. Adv Anat Pathol.

[3] Rosti G, Secondino S, Necchi A, Fornarini G, Pedrazzoli P (2019). Primary mediastinal germ cell tumors. Semin Oncol.

[4] Nogales FF, Preda O, Nicolae A (2012). Yolk sac tumours revisited. A review of their many faces and names. Histopathology.

[5] Aroor AR, Prakasha S R, Seshadri S, S T, Raghuraj U (2014). A study of clinical characteristics of mediastinal mass. J Clin Diagn Res.

[6] Mura R, Pochepnia S, Kifjak D, Khenkina N, Prosch H (2025). A diagnostic approach to mediastinal masses in clinical practice. BJR Open.

[7] Evison M, Robinson SD, Sharman A (2024). Making an accurate diagnosis of anterior mediastinal lesions: a proposal for a new diagnostic algorithm from the BTOG Thymic Malignancies Special Interest Group. Clin Radiol.

[8] Franco A, Mody NS, Meza MP (2005). Imaging evaluation of pediatric mediastinal masses. Radiol Clin North Am.

[9] Duwe BV, Sterman DH, Musani AI (2005). Tumors of the mediastinum. Chest.

[10] Ong CC, Teo LL (2012). Imaging of anterior mediastinal tumours. Cancer Imaging.

[11] Tomiyama N, Honda O, Tsubamoto M (2009). Anterior mediastinal tumors: diagnostic accuracy of CT and MRI. Eur J Radiol.

[12] Ozgun G, Nappi L (2023). Primary mediastinal germ cell tumors: a thorough literature review. Biomedicines.

[13] Takahashi K, Al-Janabi NJ (2010). Computed tomography and magnetic resonance imaging of mediastinal tumors. J Magn Reson Imaging.

[14] Juanpere S, Cañete N, Ortuño P, Martínez S, Sanchez G, Bernado L (2013). A diagnostic approach to the mediastinal masses. Insights Imaging.

[15] Nichols CR (1991). Mediastinal germ cell tumors. Clinical features and biologic correlates. Chest.

[16] Gupta S, Seaberg K, Wallace MJ (2005). Imaging-guided percutaneous biopsy of mediastinal lesions: different approaches and anatomic considerations. Radiographics.

[17] Ali AA, Abd El-Hafeez AM, Fathallah WF (2016). Yield of ultrasound-guided biopsy in anterior mediastinal lesions. Egypt J Bronchol.

[18] Petranovic M, Gilman MD, Muniappan A (2015). Diagnostic yield of CT-guided percutaneous transthoracic needle biopsy for diagnosis of anterior mediastinal masses. AJR Am J Roentgenol.

[19] Sohn A, Moran CA (2023). Primary mediastinal germ cell tumors. Semin Diagn Pathol.

[20] Kalhor N, Moran CA (2018). Primary germ cell tumors of the mediastinum: a review. Mediastinum.

[21] Huddart RA, Gabe R, Cafferty FH (2015). A randomised phase 2 trial of intensive induction chemotherapy (CBOP/BEP) and standard BEP in poor-prognosis germ cell tumours (MRC TE23, CRUK 05/014, ISRCTN 53643604). Eur Urol.

[22] Watson RA, De La Peña H, Tsakok MT (2018). Development of a best-practice clinical guideline for the use of bleomycin in the treatment of germ cell tumours in the UK. Br J Cancer.

[23] Sakurai H, Asamura H, Suzuki K, Watanabe S, Tsuchiya R (2004). Management of primary malignant germ cell tumor of the mediastinum. Jpn J Clin Oncol.

